# The Influence of Polyunsaturated Fatty Acids on the Phospholipase D Isoforms Trafficking and Activity in Mast Cells

**DOI:** 10.3390/ijms14059005

**Published:** 2013-04-25

**Authors:** Shereen Basiouni, Herbert Fuhrmann, Julia Schumann

**Affiliations:** 1 Institute of Physiological Chemistry, Faculty of Veterinary Medicine, University of Leipzig, Leipzig, An den Tierkliniken 1, 04103 Leipzig, Germany; E-Mails: basiouni@vmf.uni-leipzig.de (S.B.); fuhrmann@vmf.uni-leipzig.de (H.F.); 2 Department of Clinical Pathology, Faculty of Veterinary Medicine, Benha University, Moshtohor, Toukh, 13736 Qalioubeya, Egypt

**Keywords:** phospholipase D, polyunsaturated fatty acids, mast cells, exocytosis

## Abstract

The impact of polyunsaturated fatty acid (PUFA) supplementation on phospholipase D (PLD) trafficking and activity in mast cells was investigated. The enrichment of mast cells with different PUFA including α-linolenic acid (LNA), eicosapentaenoic acid (EPA), docosahexaenoic acid (DHA), linoleic acid (LA) or arachidonic acid (AA) revealed a PUFA-mediated modulation of the mastoparan-stimulated PLD trafficking and activity. All PUFA examined, except AA, prevented the migration of the PLD1 to the plasma membrane. For PLD2 no PUFA effects on trafficking could be observed. Moreover, PUFA supplementation resulted in an increase of mastoparan-stimulated total PLD activity, which correlated with the number of double bonds of the supplemented fatty acids. To investigate, which PLD isoform was affected by PUFA, stimulated mast cells were supplemented with DHA or AA in the presence of specific PLD-isoform inhibitors. It was found that both DHA and AA diminished the inhibition of PLD activity in the presence of a PLD1 inhibitor. By contrast, only AA diminished the inhibition of PLD activity in the presence of a PLD2 inhibitor. Thus, PUFA modulate the trafficking and activity of PLD isoforms in mast cells differently. This may, in part, account for the immunomodulatory effect of unsaturated fatty acids and contributes to our understanding of the modulation of mast cell activity by PUFA.

## 1. Introduction

Polyunsaturated fatty acids (PUFA) of the n3- and the n6-family are incorporated into cellular membranes directly or after metabolism and influence membrane properties. We have demonstrated in a previous study that the enrichment of the culture medium of C2 mast cells with α-linolenic acid (LNA), eicosapentaenoic acid (EPA), docosahexaenoic acid (DHA), linoleic acid (LA) or arachidonic acid (AA) results in an increase in the content of these fatty acids both in raft and non-raft membrane domains [1]. The PUFA-mediated modulation of the lipid composition and the physiological properties of mast cell membrane microdomains are likely to impact mast cell function. In fact, alterations in membrane lipids have already been shown to affect the exocytosis of preformed mediators (e.g., histamine) and the release of newly synthesized lipid mediators (eicosanoids) by mast cells [2,3].

The mechanism with which PUFA modulate exocytosis and vesicle fusion is not yet well understood. It is known that the enzyme phospholipase D (PLD) is involved in regulating exocytosis and vesicle trafficking [4]. PLD catalyzes the hydrolysis of phosphatidylcholine (PC), a major phospholipid of membranes, to phosphatidic acid and choline [4]. Since the ubiquitous enzyme is regulated by a variety of hormones, neurotransmitters, growth factors, cytokines, and other molecules involved in cellular communication, PLD is believed to play an important role in signal transduction of many cell types including mast cells [5]. Two mammalian PLD isoforms have been described, PLD1 and PLD2 [4]. PLD1 exhibits a variable pattern of subcellular steady-state localization. In mast cells, PLD1 co-localizes with secretory vesicles and translocates to the plasma membrane upon stimulation [6,7]. PLD2 is reported to have a different subcellular distribution, being localized predominantly at the plasma membrane regardless of cellular stimulation [4,8].

Several studies have discussed a modulating effect of PUFA on cellular PLD localization and activity. In COS-1 cells PUFA supplementation has been shown to affect the intracellular trafficking of PLD and its activity due to alterations in the membrane lipid environment [9]. In human lymphocytes a stimulatory effect of DHA and hydroxyeicosatetraenoic acid, a derivative of AA, on total PLD activity has also documented [10,11]. Other studies have demonstrated PLD-dependent effects of fatty acids in neutrophils [12], smooth muscle cells [13] and macrophages [14]. However, so far there are no data on the impact of PUFA on PLD translocation and activity in mast cells regarding the isoforms PLD1 and PLD2.

The present study was undertaken to elucidate the nature of the PUFA influence on mast cell PLD1 and PLD2 localization and activity. The enlightenment of this will further our understanding of the regulation of the exocytosis of inflammatory mediators by mast cells. The study can provide new avenues for the treatment of diseases characterized by dysregulated secretory mast cell events.

## 2. Results and Discussion

### 2.1. Distribution of PLD Isoforms in C2 Mast Cells with Regard to Stimulation Status

To elucidate the localization of PLD1 and PLD2 in mast cells, C2 cells were transiently transfected with GFP-tagged PLD1 or PLD2 fusion plasmids.

**Figure 1:**
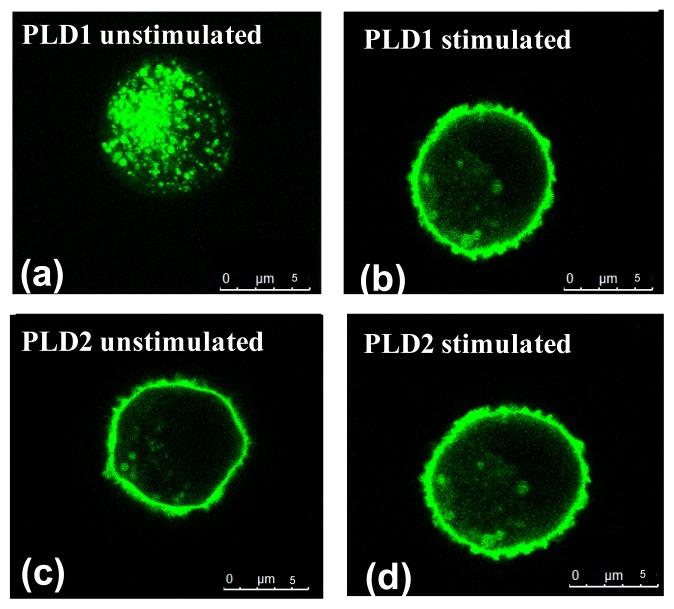
Localization of GFP-tagged PLD1 (**a** and **b**) and PLD2 (**c** and **d**) in transiently transfected C2 mast cells before (a and c) and after (**b** and **d**) stimulation with mastoparan (25 μM). Cells were transfected using Turbofect transfection reagent and analyzed 24 h after transfection. Cells were scanned by confocal microscopy (TCS SP5 STED) using a x63 oil immersion Apochromat lens (all Leica Microsystems, Mannheim, Germany). Representative figures of *N* = 3, *n* = 2 examinations are shown.

In unstimulated C2 mast cells, PLD1 was situated within intracellular vesicular structures (Figure 1a) whereas PLD2 was located at the plasma membrane (Figure 1c). For mast cell stimulation the cationic tetradecapeptide mastoparan was used [15]. After mastoparan stimulation PLD1 migrated to the plasma membrane (Figure 1b). In contrast, PLD2 localization was not affected by the stimulation (Figure 1d).

The results are in accordance with earlier studies conducted with the mast cell line RBL-2H3 [16] as well as with CHO cells [17], HL60 cells [18] and COS-1 cells [9], in which PLD1 was shown to be located intracellularly and found to translocate upon stimulation, while PLD2 was found at the plasma membrane regardless of cellular activation status.

### 2.2. Influence of PUFA on Distribution of PLD Isoforms with Regard to Stimulation Status

Using C2 transiently transfected with PLD1 or PLD2, the influence of PUFA on the distribution of PLD1 and PLD2 respectively was investigated.

**Figure 2:**
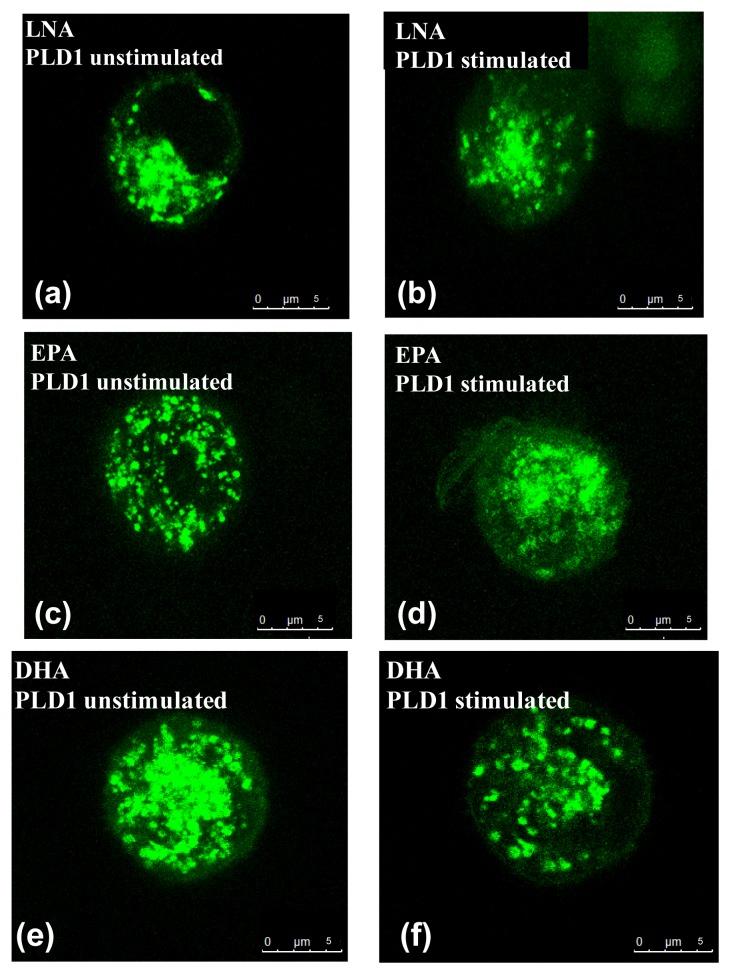

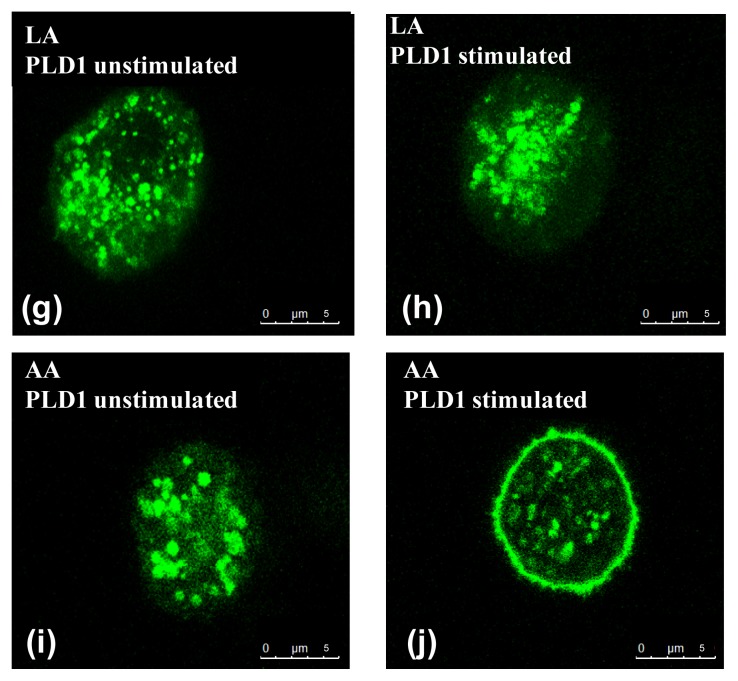
Localization of GFP-tagged PLD1 in transiently transfected C2 mast cells before (**a**,**c**,**e**,**g**,**i**) and after (**b**,**d**,**f**,**h**,**j**) stimulation with mastoparan (25 μM). Cells were transfected using Turbofect transfection reagent and analyzed 24 h after transfection. Cell culture medium was enriched for 8 days with one of the following PUFA in a concentration of 20 μmol/L: α-linoleic acid (LNA; **a** and **b**), eicosapentaenoic acid (EPA; **c** and **d**), docosahexaenoic acid (DHA; **e** and **f**), linoleic acid (LA; **g** and **h**), arachidonic acid (AA; **i** and **j**). Cells were scanned by means of confocal microscopy (TCS SP5 STED) using a x63 oil immersion Apochromat lens (all Leica Microsystems, Mannheim, Germany). Representative figures of *N* = 3, *n* = 2 examinations are shown.

We have previously shown that supplementation of C2 mast cells with LNA, EPA, DHA, LA or AA results in an incorporation of the PUFA into the cell membrane [1], and is likely to modify the composition of membrane-mediated signaling molecules as phosphatidylinositol-4,5-bisphosphate (PIP2). However, PUFA supplementation of the culture medium did not affect the intracellular localization of PLD1 in vesicular structures of unstimulated C2 mast cells in this study. We found that PUFA enrichment completely abolished the stimulator-induced translocation of the PLD1 to the plasma membrane in mastoparan-stimulated cells, with the exception of AA (Figure 2). There was no effect of PUFA supplementation on the localization of the PLD2 in either unstimulated or stimulated C2 mast cells (data not shown). In addition, no influence of the solvent used for fatty acids application (ethanol) could be observed, since both PLD isoforms behaved in a similar manner in cells cultured in medium with or without ethanol (data not shown).

The divergent effects of PUFA on PLD1 translocation found here may be due to their differing action on the translocation-mediating signaling molecules. Mastoparan activates trimeric G proteins [19,20] and increases intracellular Ca^2+^ [21,22], which is important for the activation of the protein kinase Cα (PKCα) [23]. PKCα and PLD1 are co-localized [24,25] and are translocated to the plasma membrane upon Ca^2+^-mediated activation of PKCα [26–28]. For EPA and DHA it has been shown that these PUFA inhibit the stimulation-mediated translocalization of PKCα [29,30]. As PKCα and PLD1 are translocated together, it is possible that the inhibition of PKCα by EPA and DHA leads to the inhibition of PLD1 translocation observed in our study. In contrast, AA has been described as a direct activator of PKCα [31,32] and thus does not prevent PLD1 translocation in mastoparan-stimulated mast cells. Until now, there are no data on the effects of LA and LNA on PKCα. However, based on the data presented here we suggest that LA and LNA mediate the inhibition of PLD1 translocation as EPA and DHA via a suppression of PKCα activity.

### 2.3. Influence of PUFA Supplementation on Total PLD Activity

In addition to investigating the impact of PUFA on PLD localization, we intended to explore PUFA-mediated effects on PLD activity. As an initial step, we assessed the consequences of a PUFA supplementation of C2 mast cells for eight days on total PLD activity.

**Figure 3:**
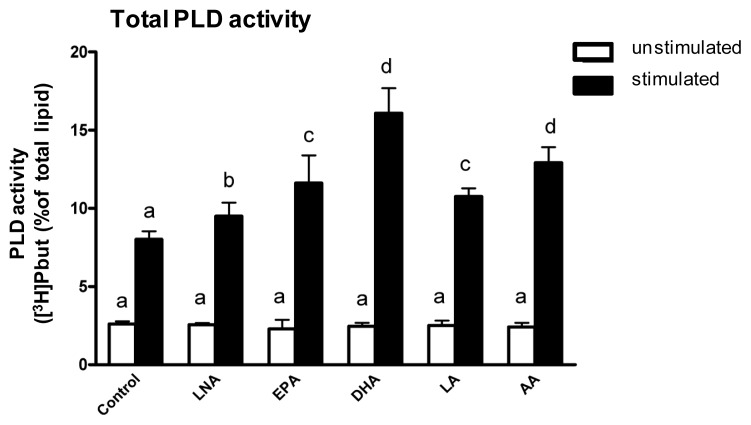
Total PLD activity of C2 mast cells. Cells were cultured for 8 days in basic medium (control) as well as in medium supplemented with 20 μmol/L of either alpha-linolenic acid (LNA), eicosapentaenoic acid (EPA), docosahexaenoic acid (DHA), linoleic acid (LA) or arachidonic acid (AA). Cells were labeled with [^3^H]myristic acid for 24 h and stimulated with mastoparan (25 μM) in the presence of 0.5% n-butanol for 30 min. Data are mean ± SD (*N* = 3, *n* = 4). Bars of one color denoted by different letters are significantly different.

Total PLD exhibited low basal activity in unstimulated C2 mast cells regardless whether and which PUFA was supplemented (Figure 3). Stimulation of the mast cells with mastoparan significantly increased the total PLD activity (Figure 3). The stimulation-induced increase in total PLD activity was evident with and without PUFA supplementation (Figure 3). However, enrichment of the C2 mast cells with LNA, EPA, DHA, LA and AA respectively further increased the total PLD activity of the cells (Figure 3). At this, the PUFA-mediated increase in total PLD activity positively correlated with the number of double bonds of the supplemented fatty acids.

Our data are in accordance with previous studies using COS-1 [9] cells and peripheral blood mononuclear cells [10], which also documented that PUFA can increase the stimulation-induced total PLD activity. However, the data obtained in the present study demonstrate for the first time that PUFA enhance total PLD activity in mast cells. Thus, we provide first evidence that the modulation of mast cell signaling, vesicular trafficking and membrane transport by PUFA [2,3] might be at least in part due to a PUFA-mediated increase of total PLD activity. It is interesting to note that despite the divergent actions of AA and the other PUFA tested on PLD1 translocation there were similar effects of all PUFA tested on total PLD activity. This supports the notion that PLD translocation and PLD activity may not be linked to one another other. The most likely cause for this can be seen in the divergent mediators, which bring about PLD1 translocation and total PLD activity. Note that the stimulation of total PLD activity is under the control of the ADP-ribosylation factor (ARF), the GTPase Rho, the phospholipid phosphatidylinositol 4,5-bisphosphate (PIP2) as well as the PKCα [8,33] whereas PLD1 translocation is mediated by PKCα only [24,25].

### 2.4. Dose Response Curves for PLD1 and PLD2 Inhibitors

**Figure 4:**
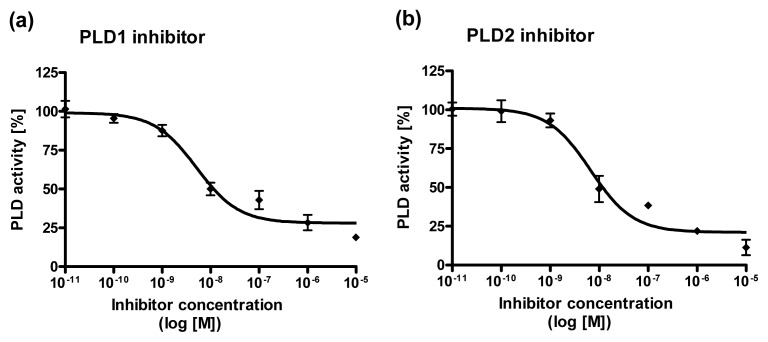
Dose response curves for PLD1 and PLD2 inhibitors. C2 mast cells were treated with different concentrations of a PLD1 (**a**) or a PLD2 (**b**) inhibitor. Cells were labeled with [^3^H] myristic acid for 24 h and stimulated by mastoparan (25 μM) in the presence of 0.5% *n*-butanol for 30 min. Data are mean ± SD (*n* = 3).

To find out which of the two PLD isoforms is implicated in the PUFA-mediated increase in total PLD activity, PLD activity was measured in the presence of specific PLD1 or PLD2 inhibitors (VU0155069 [34] and VU0364739 HCL [35], respectively). However, since the inhibitors have not been used in C2 mast cells so far, a dose response curve of each inhibitor was determined. In accordance to the literature for C2 mast cells a selective inhibition of PLD1 and PLD2 by VU0155069 and VU0364739 HCL was found (Figure 4). The recommended dose of either PLD1 or PLD2 inhibitors resulted in a nearly 60% inhibition of PLD1 or PLD2 in our mast cell line (Figure 4).

### 2.5. Influence of PUFA Supplementation on the Activity of PLD Isoforms

Using the PLD2 inhibitor VU0364739 HCL or the PLD1 inhibitor VU0155069 the influence of DHA and AA on the activity of the PLD isoforms PLD1 and PLD2 was determined.

**Figure 5:**
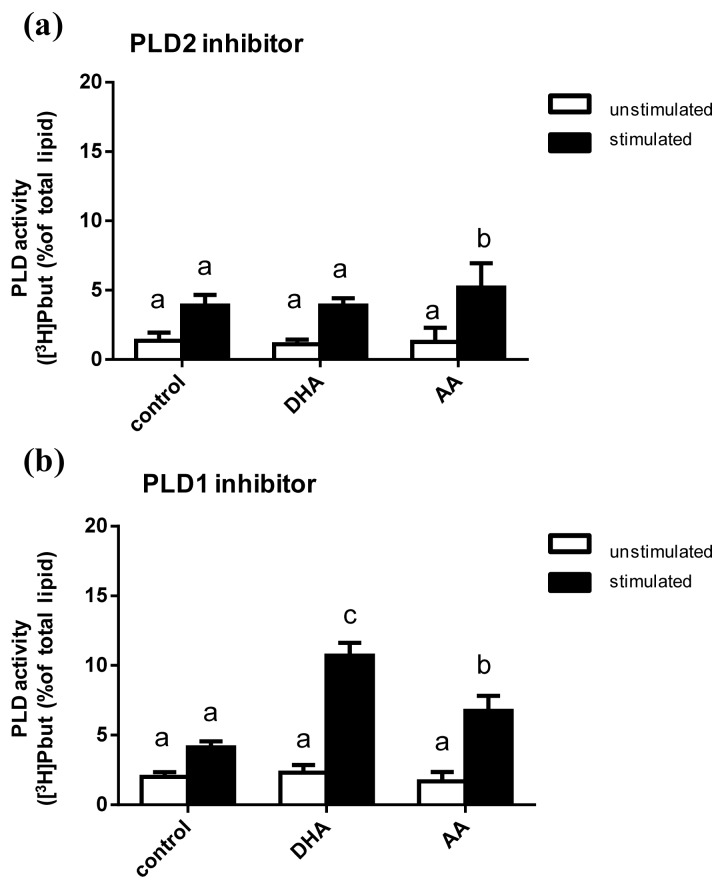
PLD activity of C2 mast cells in presence of a PLD2 (**a**) or a PLD1 (**b**) inhibitor. Cells were cultured for 8 days in basic medium (control) as well as in medium supplemented with 20 μmol/L of either alpha-linolenic acid (LNA), eicosapentaenoic acid (EPA), docosahexaenoic acid (DHA), linoleic acid (LA) or arachidonic acid (AA). Cells were labeled with ^3^ [H]myristic acid for 24 h and stimulated by mastoparan (25 μM) in the presence of 0.5% *n*-butanol for 30 min. Data are mean ± SD (*N* = 3, *n* = 4). Bars of one color denoted by different letters are significantly different.

No PUFA effects on unstimulated mast cells were observed in presence of the inhibitors (Figure 5). Stimulation of the C2 mast cells with mastoparan resulted in a significant increase in PLD activity in presence of both the PLD2 inhibitor and the PLD1 inhibitor (Figure 5). However, different effects of PUFA supplementation on stimulated mast cells could be seen. Remarkably, the decrease in PLD activity in the presence of the PLD2 inhibitor was significantly less with AA in comparison to DHA supplementation (Figure 5a). In presence of the PLD1 inhibitor the decreasing effect on PLD activity was diminished by both PUFA examined (Figure 5b).

The differential impact of AA and DHA in the presence of either a PLD2 or a PLD1 inhibitor may be due to the different activation factors of PLD1 and PLD2. Besides the common activators of both PLD1 and PLD2, namely ARF and PIP2, there are also isoform-specific PLD regulatory proteins [4]. PLD1 is selectively activated by the GTPases Rho, Rac1 and Cdc42 as well as the PKCα [4]. PLD2 is selectively activated by the GTPase Rac2 as well as the Src kinase [4]. Studies have shown that AA mediates the activation of ARF, Rho and PKCα [36,37]. This might explain the diminishing effect of AA on PLD inhibition in presence of both PLD inhibitors. For DHA the situation is different, however. On the one hand, DHA has been reported to promote ARF [11]. On the other hand, DHA has been demonstrated to down-regulate the expression of Rho and to inhibit PKCα [38,39]. In addition, it is well documented that DHA induces a significant movement of Src out of the lipid rafts thereby promoting PLD2 activity [40]. Presumably, the divergent effects of DHA on PLD1-activating mediators complement each other and lead to a diminishing effect of DHA on the inhibitor-induced decrease in PLD activity in presence of a PLD1 inhibitor only.

## 3. Experimental Section

### 3.1. Materials

All chemicals and reagents were obtained from Sigma–Aldrich (Taufkirchen, Germany) unless noted otherwise. Cell culture flasks were purchased from Greiner Bio-One (Frickenhausen, Germany). Dulbecco’s modified Eagle’s medium /HAMs F12 1:1 containing 0.14 μmol/L LA was obtained from Biochrome KG (Berlin, Germany) and Rotiszint® eco plus from Carl Roth (Karlsruhe, Germany). Silica gel 60 TLC plates were purchased from Merck Millipore (Darmstadt, Germany). [^3^H]Myristic acid was purchased from PerkinElmer LAS GmbH (Rodgau, Germany).

### 3.2. Cell Culture

The permanent canine mastocytoma cell line C2 was used. The cell line stems from isolated canine mastocytoma tumor cells, which were passaged serially 30–50 times in athymic nude mice [41,42]. The characteristics of the cell line used are comparable with other mast cell preparations like human lung mast cells, peritoneal or bone marrow-derived mast cells of rats [41,42]. 1.25 × 10^7^ cells were cultured in 75 cm^2^ flasks using DMEM/HAM’s F12 (1:1) medium supplemented with 2 mM glutamine, 25 mM HEPES (pH 7.4), 1.6 mM histidine, 100 U/mL penicillin, 100 μg/mL streptomycin, 5% FCS and 5 μmol/L α-tocopherol. To investigate the influence of PUFA, one of the following fatty acids was included in the culture medium at a concentration of 20 μmol/L using ethanol as a vehicle: α-linolenic acid (C18:3n3, LNA), eicosapentaenoic acid (C20:5n3, EPA), docosahexaenoic acid (C22:6n3, DHA), linoleic acid (C18:2n6, LA) or arachidonic acid (C20:4n6, AA) (all Biotrend, Köln, Germany). Final ethanol concentration was 0.1% *v*/*v*. Cells cultured in medium without PUFA supplementation but containing 0.1% ethanol were used as control. Cells were incubated for 8 days at 37 °C and 5% CO_2_ in a humidified atmosphere.

### 3.3. Transient Transfection

Transient transfection of C2 mast cells with pEGFP-hPLD1 and pEGFP-mPLD2 green fluorescent fusion plasmids respectively (kindly supplied by Prof. Shamshad Cockroft, Department of Physiology, University College London) was performed using chicken egg white coated 12-well plates as previously described [43]. Briefly, 22 mm^2^ surface area/well were covered with chicken egg white (0.75 mL/well) and carefully heated on a heat block at 58 °C. After 60 min, the egg white became semi-solid and adhered to the bottom of the wells. Excess egg white was washed out using PBS. The C2 cells were seeded at 5 × 10^5^ cells/well and transfected with Turbofect transfection reagent (Fermentas, Sankt Leon Rot, Germany) according to the manufacturer’s instructions and incubated for 24 h before analysis.

### 3.4. Confocal Microscopy

Evaluation of PLD1 and PLD2 localization was performed using a TCS SP5 STED microscope (Leica Microsystems, Mannheim, Germany). GFP was excited at 488 nm, and green fluorescence was detected at 595 nm. Data analysis was performed using the Leica confocal software LeicaLight version 2.61 (Leica confocal software: Mannheim, Germany, 2008).

### 3.5. Measurement of PLD Activity

PLD activity was assessed by measuring the PLD reaction product [^3^H]phosphatidylbutanol (PBut) as described by Sarri and co-workers [16] with some modifications. Briefly, C2 cells were seeded at 6 × 10^5^/well in 12-well plates and were labeled with [^3^H]myristic acid for 24 h at 37 °C in DMEM medium. Afterwards [^3^H]myristic acid was washed out and the cells were stimulated with mastoparan (25 μM) in Hepes buffer in the presence of 0.5% n-butanol for 30 min. Stimulation was terminated by adding 0.5 mL of ice-cold methanol/HCl (98:2, *v*/*v*). Cells were lysed with 500 μL methanol. Two phases were generated by adding 1 mL of chloroform and 1 mL of water. After vortexing, 900 μL were collected from the chloroform phase, and the chloroform was evaporated with a gentle nitrogen stream. Remaining lipids were solved in 20 μL of chloroform and spotted on a silica gel 60 TLC plate. Lipids were separated with the solvent system chloroform/methanol/acetic acid/water (9:1:0.3:0.04, *v*/*v*). The lanes corresponding to the PLD reaction product [^3^H]PBut were identified with authentic standards after iodine staining, scraped and counted for radioactivity. Data were expressed as the ratio of [^3^H]PBut to [^3^H]total lipids.

### 3.6. Selective Inhibition of PLD Isforms

C2 cells were labeled with [^3^H]myristic acid as described above. Labeled cells were stimulated with mastoparan (25 μM) in the presence of the PLD1 inhibitor VU0155069 (Cayman Chemical Company, Ann Arbor, MI, USA) or the PLD2 inhibitor VU0364739 HCL (Tocris Bioscience, Bristol, UK) in HEPES buffer in the presence of 0.5% *n*-butanol for 30 min. Concentrations of inhibitors used were a) 10^−11^–10^−5^ M for dose response curves b) 11 nM for the PLD1 inhibitor and 20 mM for the PLD2 inhibitor for determination of PUFA effects on PLD isoform activity. Measurement of PLD activity was performed as described above.

### 3.7. Statistical Analysis

Data are shown as means with standard deviation (SD). A two-way analysis of variance followed by unpaired Students *t* test was used to identify significant differences between means. The statistical analysis was carried out with GraphPad Prism 4 (GaphPad Software, La Jolla, CA, USA). In all cases *p* < 0.05 was assumed to indicate significant differences. The transient transfection was performed in duplicates in three independent experiments. The measurement of total PLD activity as well as the selective inhibition of PLD isoforms was performed in quadruples in three independent experiments.

## 4. Conclusions

Although phospholipases and PUFA have been known to play a crucial role in exocytosis for several decades, we are still lacking a complete picture of their mode of action. The present study showed that PUFA differentially modulate the trafficking and the activity of PLD isoforms in mast cells. All PUFA examined, except AA, prevented the translocation of the PLD1 within intracellular vesicular structures to the plasma membrane. For PLD2 no effects of PUFA on trafficking could be observed. Moreover, PUFA supplementation resulted in an increase of mastoparan-stimulated total PLD activity. At this, both DHA and AA diminished the inhibition of PLD activity in the presence of a PLD1 inhibitor but only AA diminished the inhibition of PLD activity in the presence of a PLD2 inhibitor. These differential effects of PUFA may, in part, account for the immunomodulatory effect of these fatty acids and contribute to our understanding of the PUFA-mediated modulation of mast cell activity.
